# Additional Stroke Risk Factors Beyond the CHA_2_DS_2_-VA Score in Non-Valvular Atrial Fibrillation: An Interdisciplinary Expert Opinion

**DOI:** 10.3390/jcm15051758

**Published:** 2026-02-26

**Authors:** Ersin Çagrı Simsek, Sena Sert Sekerci, Murat Gucun, Seher Tanrikulu, Emine Dundar Ahi, Begum Ozdengulsun, Dursun Aras

**Affiliations:** 1 Cardiology Clinic, Health Sciences University, İzmir Tepecik Education and Research Hospital, 35120 İzmir, Türkiye; 2 Cardiology Clinic, Altunizade Acıbadem Hospital, 34662 İstanbul, Türkiye; senasert@live.com; 3 Nephrology Clinic, Koşuyolu High Specialization Education and Research Hospital, 34865 İstanbul, Türkiye; gcn_2001@yahoo.com; 4 Department of Endocrinology and Metabolism, Acıbadem Ataşehir Hospital, 34750 İstanbul, Türkiye; tanrikuluseher@gmail.com; 5 Primary Care Medical Team, Pfizer Türkiye, 34394 İstanbul, Türkiye; emine.ahi@pfizer.com (E.D.A.); begum.ozdengulsun@pfizer.com (B.O.); 6 Department of Cardiology, Medipol University Hospital, 34196 İstanbul, Türkiye; ddaras@gmail.com

**Keywords:** anticoagulation, atrial fibrillation, ischemic stroke, prevention, cardiometabolic risk factors, obesity, chronic renal insufficiency

## Abstract

**Background/Objectives**: Comorbidity and risk factor management and avoiding stroke are pillars of optimal atrial fibrillation management. In this article, the latest literature on additional stroke risk factors not included in the CHA_2_DS_2_-VA score is reviewed in patients with non-valvular atrial fibrillation (NVAF). The possible clinical impacts of the additional risk factors in NVAF patients with a CHA_2_DS_2_-VA score of 1 are compiled with insights in an interdisciplinary panel discussion to refine daily clinical practice. **Methods**: The panel was composed of three cardiologists, one endocrinologist, and a nephrologist. The panel members finalized twenty open-ended questions covering major problems in stroke risk stratification in NVAF patients with a score of 1. The shortcomings of this clinical-based model for stroke prevention and its possible outcomes were discussed with evidence-based recommendations. The meeting outcomes are addressed for daily clinical adaptation in the present article. **Results**: Recent evidence suggests that the CHA_2_DS_2_-VA score may have shortcomings in a striking rate of NVAF patients with a score of 1. The growing body of evidence suggests that combining clinical, laboratory, and imaging predictors with the CHA_2_DS_2_-VA score may further refine stroke risk prediction and aid in decision-making for anticoagulation of NVAF patients with a score of 1. **Conclusions**: The interdisciplinary expert panel offered several recommendations based on the assessment of additional risk factors, which may enable clinicians to identify individualized patient characteristics and early multidisciplinary prevention of disease progression and personalized improvements in the long-term cardiovascular outcomes in NVAF patients with a CHA_2_DS_2_-VA score of 1.

## 1. Introduction

Atrial fibrillation (AF) is the most common cardiac arrhythmia in adults, with prevalence increasing from <1% in individuals younger than 40 years to 10–17% in those aged >80 years [1,2]. Global AF prevalence increased by 33% between 1997 and 2017, with projections indicating a further 60% rise by 2050 [2].

Non-valvular AF (NVAF) increases stroke risk 5-fold [3]. The 2024 European Society of Cardiology (ESC) AF guidelines updated the management paradigm shift from the ABC (AF Better Care) pathway established in the 2020 guidelines to the comprehensive AF-CARE framework [4,5]. This new framework encompasses four key pillars: [C] Comorbidity and risk factor management, [A] Avoidance of stroke and thromboembolism, [R] Reduction in symptoms through rate and rhythm control, and [E] Evaluation with dynamic reassessment. Notably, the updated guidelines emphasize comorbidity and risk factor management as the foundational element of optimal AF care, as well as inclusive management of AF [4,6].

NVAF patient characteristics vary across countries. The local data from a population-based study, the Turkish Atrial Fibrillation (TRAF) from 2008 to 2012, pointed out that the estimated prevalence of AF and the mortality rate at 1 year were 1.08% and 7.04%, respectively [7]. The national data from the GARFIELD-AF registry revealed that 43.3% of patients were under 65 years old, with cardiac comorbidities and prior systemic embolization [8]. Female predominance among the Turkish NVAF patients is attributed to high rates of obesity, metabolic syndrome, and cardiovascular (CV) diseases in women over 40 [7,8]. The GARFIELD-AF registry documented an overall mortality of 6.8/100 person-years in Türkiye [8], with higher mortality rates, female-to-male ratio, and proportion of patients under 65 compared to the global data.

The accurate estimation of thromboembolic risk in NVAF patients is the most important step in determining the strategy for stroke prevention in AF (SPAF) [9]. Among more than 20 risk prediction models for incident AF in the community, the CHA_2_DS_2_-VASc scoring (congestive heart failure, hypertension, age ≥75, diabetes, prior stroke or transient ischemic attack [TIA] or thromboembolism [TE], vascular disease, age 65–74, and sex category-female), a refined form of CHADS_2_, is currently the most commonly employed one in daily clinical practice [10,11].

In 2024, the CHA_2_DS_2_-VA score was proposed in the latest ESC guidelines for the management of AF to simplify the CHA_2_DS_2_-VASc score by removing the sex category (female sex is an age-dependent stroke risk modifier rather than a risk factor *per se*) [4].

According to the current ESC guidelines for AF, while patients with a CHA_2_DS_2_-VA score of 2 or more are strongly recommended/indicated to initiate oral anticoagulation (OAC) (IC), those with a score of 1 should be considered for OAC (IIaC) [4]. Patients with AF accompanied by hypertrophic cardiomyopathy (HCM) or cardiac amyloidosis are recommended to initiate OAC regardless of the risk score (IB) [4].

Additional risk factors that may influence stroke risk but are not included in the CHA_2_DS_2_-VA risk scoring system are detailed in guidelines and opinion papers developed by international societies such as the American College of Cardiology (ACC), the ESC Working Group on Cardiovascular Pharmacotherapy, and the ESC Council on Stroke [10,11]. In 2019, the ESC Working Group published an opinion paper focused OAC in NVAF patients with a CHA_2_DS_2_-VA score of 1 [11]. Obesity, proteinuria (>150 mg/24 h or equivalent), glomerular filtration rate (GFR) < 45 mL/h, N-terminal pro-B-type natriuretic peptide (Nt-proBNP), positive cardiac troponin T and I, enlarged left atrial volume (≥73 mL) or diameter (≥4.7 cm), left atrial appendix (LAA) emptying velocity (<20 cm/s), and ABC (age/biomarker/clinical history) score were specified as additional factors for thromboembolic risk modification [11]. The 2023 ACC guidelines listed a higher AF burden/long duration, AF types (persistent/permanent vs. paroxysmal), obesity, HCM, poorly controlled hypertension, GFR, proteinuria, and enlarged left atrial volume or diameter as additional risk factors, which would increase stroke risk [10].

The management of these factors requires a multidisciplinary approach. Therefore, the present study was planned to provide an interdisciplinary panel discussion on additional risk factors affecting stroke risk in NVAF patients. The panel comprised five specialists with complementary expertise: one cardiologist serving as General Secretary of the Turkish Society of Cardiology with international recognition in cardiac arrhythmias; two cardiologists with specialized knowledge in cardiac electrophysiology/arrhythmias and cardiac imaging, respectively; one endocrinologist with extensive clinical experience in diabetes and obesity management; and one nephrologist from a tertiary cardiology center with expertise in managing renal disease in cardiovascular patients.

The topics addressed in this expert panel were selected based on additional risk factors not incorporated in the CHA_2_DS_2_-VA score but clinically relevant ones in daily practice. The discussed additional risk factors are also recognized by the ESC and the ACC guidelines as potential modifiers of stroke risk in patients with atrial fibrillation. Panel members were invited based on their established scientific contributions and extensive clinical experience in the relevant domains, ensuring appropriate expertise for each topic area. The panel development process followed a structured methodology. Before the panel meeting, a comprehensive set of predefined questions was developed collaboratively with input from all participating experts. A formal panel meeting was subsequently convened to address these questions systematically. Each topic was examined in detail through structured expert discussions, enabling a thorough appraisal of the evidence and the formulation of recommendations.

The present study aims to identify and compile expert-based recommendations on frequently overlooked aspects of decision-making for OAC therapy for personalized stroke prevention in patients with NVAF and a CHA_2_DS_2_-VA score of 1, and to provide practical, easily implementable insights for treating physicians. Additionally, it seeks to raise awareness among clinicians of the clinical relevance of additional risk factors not included in the CHA_2_DS_2_-VA score of 1, as well as currently available laboratory and imaging predictors that are not yet included in stroke risk assessment in this subset of patients with NVAF. Ultimately, the study aims to contribute to improved overall disease management with a reduction in stroke risk.

## 2. Heterogeneity of Stroke Risk in NVAF Patients

Predicting AF-related ischemic stroke presents a significant clinical challenge, necessitating individualized assessment to balance the therapeutic benefits and bleeding risks of oral anticoagulant therapy, as well as non-pharmacological preventive strategies before overt anticoagulation [6]. Moreover, lately, the advent of new technologies enables clinicians to diagnose incidental nascent and subclinical AF cases more frequently [6]. However, in the present study, a subset of NVAF patients with a CHA_2_DS_2_-VA score of 1 is reviewed.

Growing evidence demonstrates that patients with a CHA_2_DS_2_-VASc score of 1 or those exhibiting distinct AF clinical phenotypes may benefit from enhanced risk stratification beyond conventional scoring systems. This comprehensive approach incorporates additional clinical risk factors, laboratory and imaging biomarkers, and phenotype-driven comorbidity management strategies [10,12,13,14,15,16,17,18,19,20,21,22,23]. These additional risk factors have been categorized within this framework to maintain a comprehensive approach; however, this classification does not directly inform anticoagulation initiation.

**Figure 1:**
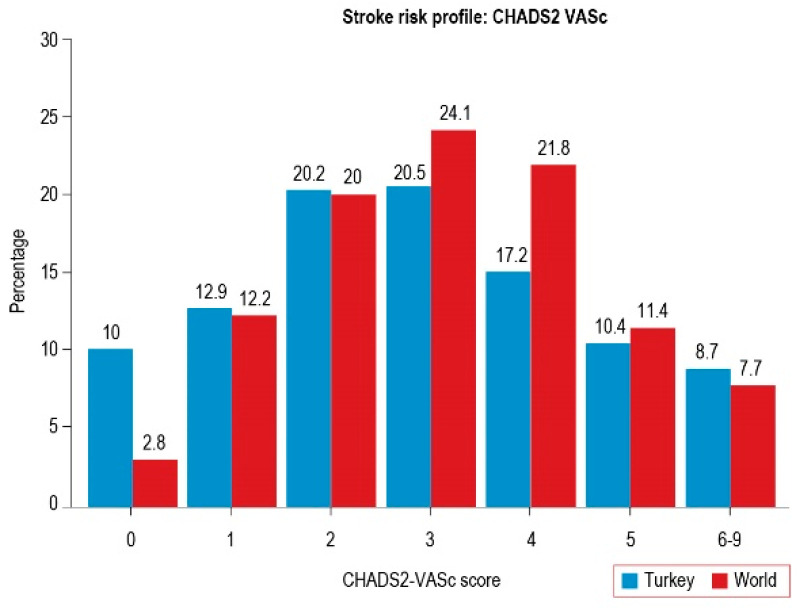
Comparison of the CHA_2_DS_2_-VASc scores in Türkiye and the world population [8].

The so-called “gray area” patients a risk score of 1, constitute 15% of NVAF patients [11]. The Global Anticoagulant Registry in the FIELD (GARFIELD) registry reported that 2.9% of patients had a score of 0, 12.7% had a score of 1, and 84.4% had a score of ≥2 [24]. Similar to the GARFIELD registry, in the real-world settings in Türkiye, 10–15% of NVAF patients are in the intermediate-risk group (a score of one), and up to 77% are in the high-risk group (a score of ≥2) (Figure 1) [8].

Based on global and local trial data, patients with intermediate risk constitute a significant subset of NVAF patients in daily practice (according to the GARFIELD registry, approximately one-sixth of high-risk patients in Türkiye) (Figure 1). The 2020 ESC AF guideline reported that many initially low-risk patients (>15%) would have ≥1 non-sex CHA_2_DS_2_-VASc risk factor at 1 year after incident AF, and 90% of new comorbidities were evident at 4.4 months after AF was diagnosed [5]. As is known, increasing age, different blood pressure control, heart failure, and incident risk factors all contribute to a dynamic change in stroke risk. Therefore, these patients should be re-evaluated for bleeding and stroke risk as well as newly added risk factors, in short intervals.

The CHA_2_DS_2_-VA score is a point-based score that assesses the differences between the included risk factors, yet it falls short in discriminating their severity. For example, the stroke risk in heart failure may differ in patients with a reduced ejection fraction (HFrEF) and those with a preserved ejection fraction (HFpEF). While a 65-year-old patient and a 74-year-old patient would be assigned a score of one for age, the stroke risks would obviously not be equal.

In a systematic literature review performed on a large NVAF cohort (21,193 from Taiwan and China and 15,837 patients from Sweden, Denmark, and France) with the CHA_2_DS_2_-VASc score of 1, it is reported that not all risk factors included in the CHA_2_DS_2_-VASc score have an equal weight on TE development; however, they are ranked in decreasing order as age 65 to 74 years, presence of diabetes, congestive heart failure (CHF), and hypertension, irrespective of sex [21]. Authors have emphasized that risk factors not included in the CHA_2_DS_2_-VA score may have a greater impact on stroke risk than expected. Contrary to the assumption that most risk factors computed in the risk prediction models are equal, studies have revealed individual differences [25].

It is noteworthy that, for example, the “true stroke risk” determination of a 65-year-old NVAF patient with borderline hypertension or at the pre-diabetic stage should include a comprehensive approach, including further assessments of clinical conditions (i.e., renal function, obesity), imaging methods (i.e., left atrial appendage morphology), and biomarker testing (i.e., Nt-proBNP) within rather shorter intervals (4–6 months).

## 3. Clinical Predictors and NVAF

### 3.1. Chronic Kidney Disorder and NVAF

According to the 2017 findings of the Global Burden of Disease, Injuries, and Risk Factors Study (GBD), the global prevalences of CKD and AF are 9.1% and 0.51%, respectively [26,27]. Patients with CKD have an increased risk of developing AF (i.e., the risk of AF development is estimated to be 20-fold higher in dialysis patients than in individuals in the general population) [28]. Observational studies have confirmed that while patients with CKD have a 47% increased risk of developing AF, patients with AF have a 64% increased risk of developing CKD [29]. Due to shared risk factors and pathogenetic mechanisms, concomitance of CKD and AF is common in daily practice, leading to challenges in clinical management and increased CV and all-cause mortality rates [29,30].

#### 3.1.1. Proteinuria

The incidence of both venous thromboembolism (VTE) and arterial thromboembolism (ATE) is higher in patients with nephrotic syndrome compared to the general population [31,32]. In patients with nephrotic syndrome, hemostatic abnormalities that can lead to a state of hypercoagulation have been identified, including urinary loss of antithrombin III leading to acquired antithrombin deficiency; decreased levels of natural anticoagulants such as plasminogen and proteins C and S (due to urinary losses); increased platelet activation; hyperfibrinogenemia; inhibition of plasminogen activation; and the presence of high molecular weight fibrinogen moieties in circulation [33,34].

Data from the ROCKET-AF (rivaroxaban once-daily, oral, direct Factor Xa inhibition compared with vitamin K antagonism for prevention of stroke and embolism trial in atrial fibrillation) and the ATRIA (anticoagulation and risk factors in atrial fibrillation) trials and the opinion paper of ESC on patients with NVAF and a CHA_2_DS_2_-VASc score of one underline the use of GFR levels in weighing the individual stroke risk (excluding patients with a GFR < 15 mL/min) [11,35,36].

In a population-based prospective study, it is reported that AF incidences were increased 1.6-fold and 3.2-fold in patients with stage 3 and stage 4 CKD, respectively, compared to individuals with normal eGFR [37]. Moreover, AF incidence is increased 3.2-fold with macroalbuminuria and 2-fold with microalbuminuria.

#### 3.1.2. GFR < 45 mL/min

Decreased eGFR increases the stroke risk in AF [27]. For every 10 mL/min/1.73 m^2^ decrease in GFR, the stroke risk increases by 7%, indicating a stepwise linear correlation [38]. Data from the ROCKET-AF and the ATRIA trials elucidated that GFR < 45 mL/min or every 10 mL/min decrease in GFR and proteinuria were potent predictors for increased stroke risk and systemic embolism in the AF cohort [35,36].

The large, contemporary, prospective, multicenter study across European countries, the EURObservational Research Programme in AF (EORP-AF) General Long-Term Registry, indicated a higher incidence of ischemic stroke in addition to the composite outcomes (major bleeding, acute coronary syndrome [ACS], and all-cause death) in a 2-year follow-up period in patients with NVAF and impaired renal function [39]. In addition, NVAF patients with a GFR < 30 mL/min/1.73 m^2^ were reported to have worse composite outcomes of thromboembolism, major bleeding, ACS, and all-cause death. A recent 24-month observational cohort study on chronic hemodialysis patients reported that the incidence of any bleeding and thrombotic events was significantly higher among patients with NVAF undergoing hemodialysis (Table 1) [40].

While 95% of patients with an eGFR < 60 mL/min/1.73 m^2^ have a CHA_2_DS_2_-VASc score ≥ 2, the ratio is increased to 99% in patients with an eGFR < 30 mL/min/1.73 m^2^ [41]. Moreover, it is determined that the annual rate of cerebrovascular and other systemic thromboembolic events is higher in the CKD patient subset with a CHA_2_DS_2_-VASc score of 0 to 1 than in those without CKD (2.9% vs. 0.2%) [41]. Therefore, the current Kidney Disease: Improving Global Outcomes (KDIGO) guideline recommends OAC for stroke prevention in all patients with decreased eGFR and AF [27].

In patients with CKD who are considered for stroke prevention due to NVAF, the first step in management is to calculate the eGFR. Many formulas have been proposed for this purpose. Although most nephrologists prefer the latest CKD-EPI equation to diagnose and classify CKD, there is no consensus on the best formula to use to guide drug dose selections [42].

Although vitamin K antagonists (VKA) are the most experienced agents, the narrow treatment range, interaction with drugs and nutrients, and the need for frequent and close monitoring make using these agents difficult for both the patient and the physician [38,42]. Unlike other patient groups, increased vascular calcification and anticoagulation-related nephropathy are seen at a higher rate compared to other new oral anticoagulants (NOACs), especially in advanced CKD patients treated with VKA. Moreover, time in the International Normalized Ratio (INR) target range for NOACs is lower in patients with advanced CKD and NVAF treated with VKA than in those without CKD or with mild CKD [38]. Thus, low time in the INR target range is associated with an increased risk for stroke, bleeding, and death in this subset [43]. VKA is metabolized in the liver, and a dose reduction of approximately 20% is required, especially in patients with advanced CKD [38]. On the other hand, studies have reported that NOACs are superior to VKAs in terms of safety and efficacy in patients with mild to moderate CKD. Therefore, recently, NOACs have been considered more frequently for anticoagulation in concomitant CKD and AF than VKA. Current evidence-based guidelines recommend using NOACs over VKA in patients with stage 1–3 CKD. However, these studies and guidelines do not include patients with advanced CKD. The KDIGO 2024 guideline recommends the dosing of NOACs based solely on limited pharmacokinetic and pharmacodynamic data (i.e., there are no randomized efficacy or safety trial data assessing clinical outcomes for stroke thromboprophylaxis in AF at CKD G4–G5 [27]).

The guideline recommendations and study data are lacking for the anticoagulation of this patient subset. It is reasonable to select OAC agents with low renal clearances in the initial therapy in CKD patients [27,38]. NOACs have a different degree of renal excretion, and apixaban has the lowest rate (27%) compared to dabigatran (80%), rivaroxaban (66%), and edoxaban (50%) [44]. While receiving OAC therapy, some patients with CKD and AF may develop acute renal failure (ARF) because of the therapeutic agents used, exposure to contrast agents, and the presence of AF. Therefore, patients should be well-informed about the common symptoms of ARF and advised to seek medical attention when necessary [38,42].

Patients with end-stage renal disease (ESRD) or who undergo hemodialysis constitute a rather challenging subset for two reasons. Firstly, as CKD patients constitute a fragile subset prone to bleeding and rapid deterioration of renal function, they should be well-informed about their clinical conditions. Secondly, patients undergoing hemodialysis are generally excluded from clinical studies. Therefore, guideline recommendations are computed mainly from observational studies and, to a lesser extent, from randomized clinical trials [4,10,45,46]. In the 2023 ACC AF guideline, warfarin (INR 2.0–3.0) or an evidence-based dose of apixaban is recommended to reduce the stroke risk in NVAF patients with end-stage CKD (CrCl < 15 mL/min) or on dialysis [10]. In the KDIGO 2024 guideline, NOACs are recommended in preference to VKAs (e.g., warfarin) for thromboprophylaxis in AF patients with CKD G1–G4 (1C) [27]. It is recommended to adjust the NOAC dose according to GFR, especially with caution in CKD G4–G5 [27,44]. However, the availability of NOACs differs according to renal function capacity [4,44,47]. The KDIGO 2024 guideline recommends that low-dose apixaban (2.5 mg orally twice daily) in CKD G5/G5D may be considered to reduce bleeding risk until clinical safety data are available [27]. As there are no clear guideline recommendations on which thromboprophylactic agent to choose, employing patient education, shared decision-making, and interdisciplinary consultation with the cardiology clinic would provide the most inclusive management for CKD and AF.

### 3.2. Improving the Management of Patients with Coexisting CKD and NVAF in Routine Clinical Practice

Both CKD and AF are complex clinical conditions, and a one-sided management perspective will reduce the treatment success and increase the risk of complications when they coexist. It should be noted that the risk factors included in the CHA_2_DS_2_-VA scoring system are similar to the etiological factors of CKD. Therefore, every patient with a high score in this scoring system should be viewed as a potential CKD patient, even if there is no clinically overt renal dysfunction yet.

Bullet Summary for NVAF Patients with CKD

- NVAF patients with an index CHA_2_DS_2_-VA score of one should be assessed for renal function at baseline and in routine follow-up visits according to individual patient needs.
- The frequency of follow-up visits of CKD patients on OAC should be determined based on the patient-specific requirements and according to the disease stage.
- Time intervals between follow-up visits should be shorter in patients with advanced CKD. These patients would benefit more from close interdisciplinary collaborations between nephrology and cardiology clinics.
- Initiation or discontinuation of OAC should be a joint interdisciplinary decision-making process in CKD patients with NVAF.
- It is recommended that interdisciplinary collaboration should be initiated in patients with eGFR < 60 mL/min/1.73 m^2^ and/or proteinuria. However, considering the daily workloads of both cardiology and nephrology outpatient clinics, patients with an eGFR < 30 mL/min/1.73 m^2^ would benefit from interdisciplinary dialog.
- It should be noted that in the current AF guidelines, GFR < 45 mL/min is underlined as an additional risk factor for stroke risk, not included in CHA_2_DS_2_-VA.
- Cardiologists should consult a nephrologist when they determine a protein level of more than 200 mg in spot urine.
- NVAF patients who develop ARF during follow-up may consult with a nephrologist for precautionary purposes.
- Patients should be well-informed about the coexistence of CKD and AF diseases.
- Joint meetings should be organized to establish an interdisciplinary structure for assessing and planning treatment for patients with coexisting CKD and AF, according to current guidelines.

### 3.3. Obesity and NVAF

Based on GBD 2021 data, it is estimated that almost two out of three adults over the age of 25 years will be overweight (BMI > 25 kg/m^2^) and obese (BMI ≥ 30 kg/m^2^) by 2050 [48]. According to the Turkish Statistical Institute data, approximately 21.1% of the adult population in Türkiye is obese [49]. In a meta-analysis, the prevalence of obesity is reported as 33.2% in females and 18.2% in males, with a mean BMI of 27.4 kg/m^2^ (28.2 kg/m^2^ for women and 26.5 kg/m^2^ for men).

Obesity is a well-established risk factor for both arterial thrombosis and venous thromboembolism (VTE). Evidence from large population studies, particularly a Swedish cohort study following men born between 1945 and 1961, demonstrates that each standard deviation increase in BMI during childhood and puberty correlates with a linear increase in both VTE and arterial thrombosis in adulthood [50].

Current clinical guidelines highlight the detrimental role of obesity in AF management, largely due to its close association with cardiometabolic comorbidities such as physical inactivity, diabetes mellitus, hypertension, and tobacco use [4,10]. Moreover, major landmark trials for NOACs, such as RE-LY, ARISTOTLE, ENGAGE-TIMI 48, and ROCKET-AF, conducted on NVAF patients have revealed that approximately 30–40% of patients had obesity (BMI ≥ 30 kg/m^2^) [51,52,53,54,55].

### 3.4. Commonly Encountered Issues in NVAF Patients with Obesity

Compared to NVAF patients with normal BMI, individuals with obesity have a higher risk of AF development. Data from the Framingham Study have shown that obesity increases the risk of developing AF, with each 1 kg/m^2^ increase in BMI associated with a 4% higher risk of incident AF [10]. Moreover, each 5 kg/m^2^ increase in BMI is associated with a 10–13% increased risk of AF recurrence after ablation [4,10]. Hence, current guidelines recommend an ideal target of at least 10% weight loss for all obese individuals with NVAF as part of comprehensive risk-factor modification [4,10]. In patients with BMI ≥ 40 kg/m^2^, bariatric surgery may be considered in conjunction with lifestyle modification and medical therapy [4].

In clinical practice, obesity may be underestimated as a contributor to thromboembolic risk, as it is not directly incorporated into the CHA_2_DS_2_-VA score. Nevertheless, obesity should be regarded as a risk modifier rather than an independent risk factor. Obese patients with NVAF frequently exhibit obesity-related comorbidities such as diabetes mellitus, dyslipidemia, hypertension, obstructive sleep apnea, and cardiorenal metabolic syndrome, all of which may amplify thromboembolic risk. Therefore, thromboembolic risk assessment in obese patients with NVAF should not rely solely on CHA_2_DS_2_-VA but should incorporate a comprehensive evaluation of cardiometabolic comorbidities, particularly in individuals at the borderline of anticoagulation thresholds.

Some cross-sectional and epidemiological studies have reported that aged obese people with a particular disease may have lower mortality than their normal-weight or underweight counterparts. This is defined as “the obesity paradox.” The obesity paradox was first reported among hemodialysis patients [56]. The presence of the obesity paradox is debatable because it is believed that differences in the methodologies of the studies may have led to conflicting results. After the statistical adjustment, the values were greatly reduced.

In the elderly population, age-related changes such as sarcopenia and osteoporosis may limit the accuracy of BMI. For instance, short stature due to osteoporosis may lead to higher BMI values than they should be. Population-based studies suggest a J-shaped association between BMI and mortality among elderly individuals with chronic conditions, with no significant increase in mortality in elderly individuals with stage 1 obesity (BMI = 30–34.9 kg/m^2^), but the mortality rate increases in those with stage 2 (BMI = 35–39.9 kg/m^2^) and stage 3 (BMI ≥ 40 kg/m^2^) obesity and also in those who are underweight (BMI < 18.5 kg/m^2^). Consequently, stroke prevention strategies in this population should be guided by a dynamic, individualized risk assessment, and oral anticoagulant therapy (either VKAs or NOACs) should be tailored accordingly.

Meta-analysis performed on the obese subgroups within landmark studies for NOACs indicated that NOACs were more effective when compared to VKAs in stroke prevention, with additional safety benefits on major bleeding [57]. Data from a recent meta-analysis denoted that NOACs provided a significant reduction of 20% in the combined risk of any stroke, systemic embolism, myocardial infarction, or all-cause mortality compared to warfarin in obese patients (BMI ≥ 30 kg/m^2^) with AF [58]. In the ARISTOPHANES study, which was performed on more than 88,000 obese patients with NVAF in the United States, propensity score matching between NOACs (apixaban, dabigatran, rivaroxaban) and warfarin [59]. Apixaban and rivaroxaban had a lower risk of stroke (dabigatran had a similar risk), while apixaban and dabigatran had a lower risk of major bleeding (rivaroxaban had a similar risk) compared to warfarin. In the current ACC AF guideline, it is stated that it is reasonable to prefer NOACs over warfarin in stroke risk reduction in NVAF patients with a BMI ≥ 40 (Class III obesity) [10]. According to the ESC 2024 guideline for the management of AF, it is emphasized that there is limited evidence regarding the use of NOACs in Class III obesity and highlights the need for further prospective data [4]. Bariatric surgery is included in the holistic management of AF in Class III obesity patients when a rhythm control strategy is planned to reduce the recurrence and progression of AF. The number of studies about the efficacy and safety of NOACs is increasing in morbidly obese individuals with NVAF, yet the evidence is still unclear [57,60,61]. On the other hand, sudden and uncontrolled reductions in body weight may cause the loss of muscle mass and sarcopenia in elderly NVAF patients with obesity, which may further complicate anticoagulation management and frailty-related bleeding risk.

### 3.5. Improving the Management of Patients with Coexisting Obesity and NVAF in Routine Clinical Practice

Endocrinological diseases generally require interdisciplinary teamwork integration. Therefore, endocrinologists work in close collaboration with many medical disciplines, the leading ones being cardiology and nephrology. Since weight loss is a cornerstone in comprehensive AF management, close collaboration between endocrinology and cardiology may facilitate the achievement of individualized targets in the NVAF subset with obesity.

### 3.6. AF Type and Burden/Long Duration

There is a complex interplay between AF and comorbidities, modulating the rate of AF progression and the risk of AF-related outcomes. In addition, the type, burden, and duration of AF are clinically significant in disease management. Currently, AF burden is reported as a reliable quantitative measure of intermittent AF to estimate the attributable risk of AF, initiation of anticoagulation, and the efficacy of antiarrhythmic treatment [62,63]. However, the use of variable technological methods, such as cardiac implantable electronic devices (CIEDs), implantable loop recorders, Holter recording, and wearable devices, presents a complexity in the documentation and interpretation of AF types [62,63,64,65]. Limited-time intermittent monitoring requires a duration of at least 4 weeks of accumulated monitoring per year to be compared with continuous rhythm monitoring [62]. Nevertheless, the atrial rhythm needs to be inspected for qualification and quantification in CIEDs [64,65].

**Figure 2:**
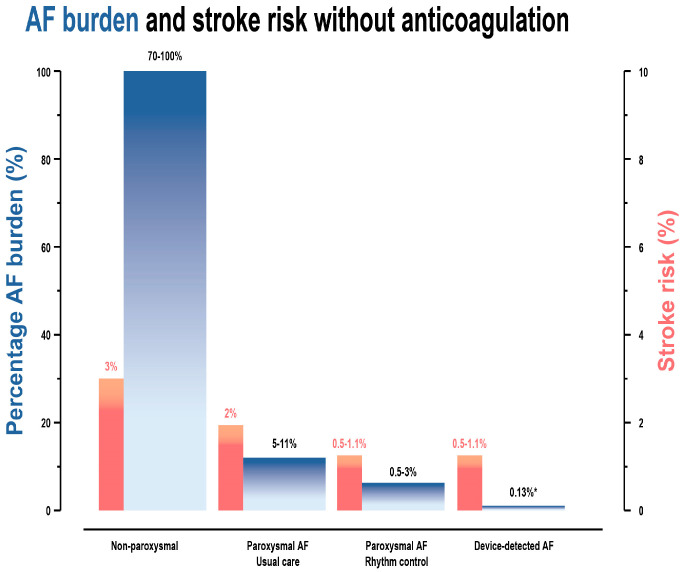
The estimated risk of stroke associated with atrial fibrillation burden or atrial fibrillation pattern. * According to the data of the LOOP study, the median atrial fibrillation burden is 0.13% (interquartile range 0.03–1.05). Atrial fibrillation burden was defined as the cumulative duration of all atrial fibrillation episodes lasting >6 min from the first adjudicated atrial fibrillation episode onward, divided by the total duration of monitoring [63].

The average AF burden is more pronounced in persistent AF (70–100%) compared to paroxysmal AF (5–11%) [66]. Even though the risk for stroke or systemic embolism is higher in patients with non-paroxysmal AF as compared with patients with paroxysmal AF, it still does not change the need for anticoagulation therapy (Figure 2) [63]. However, it is reported that CIEDs are not directly related to the clinical classification of paroxysmal, persistent, or permanent AF [64,65].

Initiating OAC in younger patients with AF can present a clinical dilemma for physicians. For instance, the thromboembolic risk profile of a 45-year-old individual experiencing a brief episode of paroxysmal AF differs substantially from that of a 65-year-old patient with the same arrhythmia. Conversely, in a 66-year-old patient presenting with a 3-h AF episode, prompt initiation of OAC is warranted. In a meta-analysis investigating the association between AF burden >5 min and increased stroke risk in 53,141 patients having CIEDs, it was reported that there was a significant linear association between increased AF burden and stroke risk (67% increased risk of stroke) [67]. Data from 1-year continuous monitoring using a dual-chamber pacemaker equipped with diagnostic features on 568 patients with symptomatic atrial tachyarrhythmias (AF absence/presence and duration), combined with the CHADS_2_ score, revealed that the discrimination between the high- and low-risk patients was improved more for thromboembolism if the evaluation of AF presence/duration was performed concomitantly with the clinical risk scoring [68].

Evidence from the prospective long-term follow-up studies with continuous ECG monitoring highlighted that a significant proportion of AF cases (38%) were diagnosed through the detection of asymptomatic episodes during rhythm monitoring [69]. The AFFIRM trial further highlighted that silent AF episodes were present at baseline in approximately 12% of patients, predominantly among males with longer AF duration, lower peak ventricular rates during AF, and preserved left ventricular function [70]. The ARTESIA trial showed that among patients with subclinical atrial fibrillation lasting 6 min to 24 h and risk factors for stroke, apixaban resulted in a lower risk of stroke or systemic embolism than aspirin but a higher risk of major bleeding [71]. Of note, the Asymptomatic Atrial Fibrillation and Stroke Evaluation in Pacemaker Patients and the Atrial Fibrillation Reduction Atrial Pacing (ASSERT) study data revealed that only 8% of the cohort had AF in the prior months, whereas most of the subjects showed AF episodes long before or only after the ischemic event [72,73].

Bullet Summary for AF Type and Burden/Long Duration

- The consensus on AF diagnosis recommends a 10 s measurement of a standard 12-lead ECG and a 30 s measurement on ECG devices with one or more leads. The exact time interval spent on diagnosing AF on monitoring devices is unclear.
- Non-ECG-based methods and devices, typically using photoplethysmography, are not diagnostic for AF but may be indicative of AF.
- Unlike subclinical AF, patients with clinically documented paroxysmal AF should receive anticoagulation treatment, preferably with a NOAC, similar to subjects with non-paroxysmal AF.
- In the 2024 ESC AF guidelines, it is recommended to consider a prolonged non-invasive ECG-based approach in individuals aged ≥75 years or ≥65 years with additional CHA_2_DS_2_-VA to ensure earlier detection of AF [4].
- Recently, there has been a paradigm shift in AF management: AF burden reduction is included in the therapeutic goals along with anticoagulation, treatment of comorbidity, and risk factor management. Consequently, AF ablation is reported to reduce AF burden, thus AF-related CV outcomes [63].

## 4. Laboratory Predictors and NVAF

### NT-proBNP and Cardiac Troponin

N-terminal pro-B-type natriuretic peptide (NT-proBNP) and cardiac troponin (cTnT) are widely available biomarkers for the diagnosis and prognosis of cardiac diseases. In a large community-based study, the association between NT-proBNP and incident AF was reported in the older adult population independently of established risk factors [74]. Cardiac troponin T (cTnT) and troponin I (cTnI) are regulatory proteins controlling the calcium-mediated interaction between actin and myosin. cTnT and cTnI are different from skeletal muscle-specific isotypes. Cardiac TnT is associated with incident AF and AF recurrence after radiofrequency ablation [75]. In the substudy of ARISTOTLE, cTnT and cTnI concentrations above the median were determined in the majority of NVAF patients with at least one risk factor, and the elevated levels were moderately correlated with increased risk of stroke and CV events over a median of 1.9 years [76]. The RE-LY biomarker substudy reported that elevated cTnI and NT-proBNP were independently related to increased risks of stroke and mortality in NVAF patients [77]. Troponin and NT-proBNP elevations are observed in 25% and 75% of NVAF patients, respectively [77]. Therefore, the evaluation of cardiac biomarker levels together with the CHA_2_DS_2_-VA risk score can provide additive prognostic information in NVAF patients with at least one risk factor for stroke.

Informed by the mechanistic pathways underlying AF pathophysiology, a diverse array of molecular biomarkers has emerged as compelling tools for stroke risk stratification and the formulation of mechanism-oriented, personalized treatment strategies. Combining the execution of cardiac biomarkers and clinical risk scoring, the ABC (age, biomarker, and clinical history) stroke risk score was proposed for risk stratification of NVAF patients [78]. The ESC Working Group published an opinion paper concerning OAC therapy in NVAF patients with a CHA_2_DS_2_-VASc score of one, and recommended the incorporation of NT-proBNP and cardiac troponins (cTnT and cTnI) into clinical decision-making frameworks for AF patients in the gray zone [11]. In the 2020 ESC AF guideline, it is stated that routine use of biomarker-based scoring has no additive benefit in decision-making for initial stroke prevention treatment in patients already qualifying for treatment based on the CHA_2_DS_2_-VASc score, as such an approach would be practically limited and increase healthcare costs [5]. Still, biomarkers could further refine stroke risk differentiation among patients initially classified as low risk and those with a single non-sex CHA_2_DS_2_-VASc risk factor [5].

High cardiac troponin or NT-proBNP indicates that the patient has a CV problem or CV aging. Ultimately, similar to CRP, they emphasize an underlying significant condition. Inclusion of cardiac biomarkers in stroke risk evaluation may provide a more dynamic and comprehensive patient assessment and also the determination of a more personalized risk stratification for gray zone patients. For example, assessing a 50-year-old patient with controlled hypertension, despite controlled hypertension, the patient will receive a CHA_2_DS_2_-VA score of one. High NT-proBNP levels will signal an impending additional clinical condition. The follow-up intervals may be shortened, and further investigations may be added to the routine follow-up parameters. If the patient’s cardiac troponin is elevated, it may be interpreted that the total CV mortality risk is increasing. Physicians can perform a dynamic and comprehensive assessment plan, including coronary computed tomography angiography (CCTA), and increase the number of control visits.

Another patient subgroup for which biomarker levels are elucidating is NVAF patients with HFpEF. The chicken or the egg causality dilemma problem is already between AF and HFpEF. Although the resolution of this dilemma is still unclear, the treatment decision guided by NT-proBNP levels is the most appropriate approach in such patients. In NVAF patients with heart failure symptoms and confirming physical examination findings, determination of NT-proBNP levels will assist in ascertaining the diagnosis [79]. Nonetheless, optimization of heart failure therapy improves hemodynamics, reduces congestion, and may lower thromboembolic vulnerability in AF populations [80].

## 5. Imaging Predictors

### 5.1. Enlarged Left Atrium (LA) Volume (≥73 mL) or Diameter (≥4.0 cm)

In daily clinical practice, the definite diagnosis of NVAF is made by the absence of mechanical heart valves and moderate to severe mitral stenosis in echocardiography. Moreover, echocardiography enables clinicians to determine additional risk factors, such as left atrial (LA) dilatation (increased risk of AF and TE), LV outflow tract obstruction (LOTO), systolic anterior movement (SAM) in the mitral valve, LV apical aneurysm, left ventricular appendix dysfunction (more clearly observed on TEE), and extent and distribution of LV hypertrophy (Table 2).

Patients with the enlarged LA on echocardiography, accompanying structural left heart diseases, signs of previous silent stroke on magnetic resonance imaging (MRI) without any history of previous stroke or transient ischemic attack (TIA), positive family history for AF, and low bleeding risk are good candidates for OAC treatment.

LA enlargement indicates diastolic burden and is a predictor of CV outcomes, such as AF, stroke, CHF, and CV death [81]. Both the 2023 ACC/AHA/ACCP/HRS Guideline for the Diagnosis and Management of Atrial Fibrillation and the 2024 ESC/EACTS Guidelines for the Management of Atrial Fibrillation emphasize that LA size, preferably assessed by left atrial volume index (LAVI), is a key marker of atrial remodeling and a powerful predictor of new-onset and recurrent AF [4,10]. According to the American Society of Echocardiography/European Association of Cardiovascular Imaging (ASE/EACVI) chamber quantification standards, which are adopted by both societies, an LAVI > 34 mL/m^2^ represents *left atrial enlargement* and independently increases the risk of AF development, recurrence after ablation or cardioversion, and adverse cardiovascular outcomes [82]. The 2023 AHA and 2024 ESC AF guidelines recommend using LAVI rather than the anteroposterior (AP) diameter because volumetric assessment accounts for the complex, non-spherical geometry of the atrium. In the 2023 ACC/AHA guideline, an enlarged LA is defined as either a LAVI > 34 mL/m^2^ or an AP diameter ≥47 mm (4.7 cm) when volumetric data are not available [10]. These thresholds are incorporated into risk assessment for AF progression, stroke, and outcomes after rhythm control interventions.

Similarly, the 2024 ESC AF guidelines adopt the same volumetric cutoff, identifying LAVI > 34 mL/m^2^ as the threshold for LA enlargement associated with increased AF incidence and recurrence [4]. The guidelines further note that larger LA size (e.g., LAVI ≥ 40–48 mL/m^2^ or AP diameter ≥ 45–50 mm) is associated with advanced atrial cardiomyopathy and poorer rhythm-control outcomes. However, no single universal “high-risk” cut-off is mandated.

In summary, both major 2023–2024 AF guidelines converge on LAVI > 34 mL/m^2^ as the primary threshold for clinically significant LA enlargement and increased AF risk, with AP diameter ≥47 mm as an alternative when 3D volume data are unavailable. Increasing LA size beyond these levels (particularly LAVI ≥ 40 mL/m^2^**)** denotes progressively higher risk for AF occurrence, recurrence, and structural remodeling. LA enlargement is represented more accurately by the LA volume than the M-mode LA dimensions.

### 5.2. Left Atrial Appendage (LAA) Morphology and Emptying Velocity (<20 cm/s)

It is reported that 15% of patients with AF have LAA thrombus, and nearly 90% of thrombi originate in the LAA in NVAF patients [83,84]. Therefore, if available, evaluation of left atrial appendage emptying velocity (LAAEV) and details about the LA morphology provide useful information about the stroke risk in NVAF patients beyond using the CHA_2_DS_2_-VA scoring alone.

Among the four morphologic shapes of LAA, the chicken wing (48%) is the most common and the one with the least association with embolus formation [83,85,86,87]. The second common one is cactus (30%), followed by windsock (19%), and lastly, the cauliflower (3%) [83,86,87]. The cauliflower-type appearance is very lobular and segmented, and the risk of embolism is high due to the variable number of lobes, short length, and irregular orifice. The morphological characteristics and the correlated stroke risks are presented in Table 3 [83,86,87]. It was reported that non-chicken wing LAA morphology increased the stroke risk more than 6-fold compared to chicken wing morphology in NVAF patients (OR 10.1, 95% CI: 1.25 to 79.7, *p* = 0.019) [87].

A transesophageal echocardiogram (TEE) is an extremely significant CV imaging modality. In NVAF patients, TEE can provide information about additional TE risks such as mitral annular calcification, as well as the morphology and functioning of the LA and the presence of thrombus in the LAA. This procedure is usually employed before cardioversion, in exclusion of LAA thrombus, and in determining the cause of emboli in post-stroke patients. Nevertheless, the 10-year retrospective analysis of the incidence of ischemic stroke in NVAF patients with confirmed LAA thrombus by using TEE revealed low stroke rates over a six-month follow-up period under optimal OAC treatment [88].

Detailed examination of LAA by using TEE enables cardiologists to classify LAAEV as normal (≥40 cm/s), moderate risk (20–40 cm/s), and high-risk (<20 cm/s) for emboli [84]. A smoky appearance, indicating slowed emptying, indicates the increased risk of embolism. Therefore, LAA closure (LAAC) is commonly encountered as a site-specific interventional therapy. Of note, OACs are the cornerstone of stroke prevention in AF, and LAAC should not be employed as a broader replacement for OAC. Nevertheless, LAAC may be preferred in NVAF patients with increased stroke risk if they are not suitable for long-term OAC.

Left Atrial Appendage Evaluation for Stroke Risk Stratification: Clinical Practice Recommendations

- LAA morphology is assessed with TEE or 3D echocardiography.
- In patients with cauliflower and windsock morphologies, more aggressive anticoagulation or LAA closure should be considered.
- Anatomical complexity and volume increase result in blood stasis and spontaneous echocontrast (SEC) in LAA, leading to thrombosis and embolism.
- The combined assessment of the morphology and emptying velocity (<20 cm/s) of LAA may help clinicians in embolic risk estimation. However, not all functional anatomical markers provide an evidence-based indication for anticoagulation, but rather may indicate an increased thermogenicity risk [88].
- LAA morphology can be combined with the CHA_2_DS_2_-VA score when assessing stroke risk, but it is not yet routinely included in the guidelines.

## 6. The Other Additional Risk Factors

### 6.1. Hypertrophic Cardiomyopathy (HCM)

Hypertrophic cardiomyopathy (HCM) is an autosomal dominant cardiac myocyte disease leading to dysfunction in the contractile machinery of the heart. In echocardiography, the left ventricular (LV) wall thickness exceeds 15 mm, which causes LV outflow tract obstruction. Despite the absence of randomized controlled trials, the 2024 ESC AF guideline recommends OAC for patients with AF in the presence of HCM or cardiac amyloidosis, regardless of the CHA_2_DS_2_-VA score (Class IB) [4]. Being a structural disease, HCM triggers AF development alone, and AF is encountered in 25% of HCM cases [89]. Coexisting AF may indicate more advanced cardiomyopathy. Since the CHA_2_DS_2_-VA score is insufficient to predict the risk of embolism in HCM, patients with coexisting HCM and AF should be considered high-risk and receive anticoagulation regardless of any score. Similar to NVAF patients with HCM, NVAF patients with cardiac amyloidosis are also recommended to initiate OAC regardless of the risk score (IB) [4].

### 6.2. Obstructive Sleep Apnea Syndrome (OSAS)

Obstructive Sleep Apnea Syndrome (OSAS) is considered an independent and potentially modifiable risk factor in the development of NVAF. OSAS causes atrial structural and electrical remodeling through intrathoracic pressure changes, hypoxemia, sympathetic activation, and systemic inflammation [90]. In recent years, there has been growing evidence that OSAS not only increases the frequency of AF but also elevates the risk of thromboembolic events [91,92]. Also, it is uncertain whether the treatment with continuous positive airway pressure (CPAP) can prevent major cardiovascular events. CPAP used in OSAS therapy reduces AF recurrence and major cardiovascular events in elderly NVAF patients with OSAS [93]. However, the SAVE (Sleep Apnea cardioVascular Endpoints) trial failed to demonstrate a difference in clinical outcomes in those randomized to CPAP therapy or placebo [94]. Therefore, screening AF patients for OSAS and initiating treatment when appropriate was not included in the current guidelines [4]. In summary, the coexistence of OSAS and AF independently increases stroke risk and may require risk stratification beyond the CHA_2_DS_2_-VA score [95].

### 6.3. Future Research Directions

The most clinically employed CHA_2_DS_2_-VA scoring system does not capture all risk factors for systemic embolism and stroke in patients with NVAF. Scoring systems that incorporate risk factors not included in the CHA_2_DS_2_-VA scoring system, such as biomarkers and chronic renal failure, are being developed but are not widely used. Even if a scoring system encompassing all risk factors were developed, it would be difficult to use in daily practice. Data suggest that using clinical, laboratory, and imaging predictors in conjunction with the CHA_2_DS_2_-VA score could further refine stroke risk prediction in patients with an NVAF score of one and have a substantial impact on decision-making for initiating anticoagulation. Since the integration of clinical, imaging, and inflammatory markers into the CHA_2_DS_2_-VA risk score is supported by large cohorts and multicenter studies, physician awareness should be increased about the additional risk factors in risk stratification for systemic embolism and stroke risk in patients with NVAF and a moderate CHA_2_DS_2_-VA score.

## 7. Conclusions

This article highlights that some NVAF patients with a CHA_2_DS_2_-VA score of one may progress to a score of two due to additional risk factors and comorbidities. In addition to the CHA_2_DS_2_-VA score, it should be emphasized that assessment of clinical, laboratory, and imaging predictors, such as obesity, CKD, NT-proBNP levels, and LAAEV, should also be integrated in the personalized decision-making for stroke prevention in NVAF patients with a score of one. To achieve better clinical outcomes for patients with a CHA_2_DS_2_-VA score of one, it is essential that treating physicians remain vigilant. This includes implementing guideline recommendations more effectively in clinical practice, fostering interdisciplinary collaboration, examining additional risk factors for each patient, and developing a dynamic follow-up and disease management plan.

## Figures and Tables

**Table 1:** Bleeding and thrombotic events by patient subgroups [40].

		All Cohort (*N* = 224)	Without AF (*n* = 181)	With AF (*n* = 43)	*p* Value
Bleeding Outcomes	Any bleeding event	80 (35.7%)	58 (32.4%)	22 (51.2%)	*0.029*
	Major bleeding	21 (9.4%)	13 (7.2%)	8 (18.6%)	*0.043*
	Clinically relevant bleeding	23 (10.3%)	20 (11.4%)	3 (7.0%)	0.61
	Minor bleeding	24 (10.7%)	19 (10.5%)	5 (11.6%)	1
	Death due to bleeding	12 (5.3%)	6 (3.3%)	6 (14.0%)	*0.016*
Thrombotic Outcomes	Any thrombotic event	60 (26.8%)	39 (21.5%)	21 (48.8%)	*0.0006*
	Ischemic stroke	15 (6.7%)	7 (3.9%)	8 (18.6%)	*0.0117*
	AVF thrombosis	28 (12.5%)	21 (11.6%)	7 (16.2%)	0.563
	MI	10 (4.5%)	5 (2.8%)	5 (11.6%)	*0.034*
	PTE	7 (3.5%)	6 (3.3%)	1 (2.3%)	1
	Death due to thrombosis	12 (5.4%)	5 (2.8%)	7 (16.2%)	*0.0016*

AVF, arteriovenous fistula; MI, myocardial infarction; PTE, pulmonary thromboembolism.

**Table 2:** Additional echocardiographic risk modifiers in NVAF patients with intermediate stroke risk (CHA_2_DS_2_-VA = 1) *.

Additional Risk Factors	When to Consider?	How to Assess?	Impact On Stroke Risk
Left atrial enlargement (≥47 mm)	When identified on TTE/TEE	2-D echocardiographic LA diameter	↑ Risk of thrombus formation and embolus
Low LAA emptying velocity (<20 cm/s)	If TEE is available	Doppler interrogation of LAA	↑ Risk of thrombus and spontaneous echo contrast
Spontaneous echocontrast (SEC)	If visible on TEE	Qualitative intensity grading (LAA)	High stroke risk, especially grade 3–4
Non-chicken wing LAA morphology	When morphology can be defined by TEE or CT	LAA morphologic classification	↑ Embolic risk in non-chicken wing types
Left ventricle EF < 50% or LV dilatation	If LV is evaluated by echocardiography	LV EF and LVEDD measurement	↑ Stroke risk in association with heart failure

LAA, left atrial appendage; TTE/TEE, transthoracic/transesophageal echocardiography; SEC, spontaneous echocontrast; LV, left ventricle; EF, ejection fraction; LVEDD, left ventricular end- diastolic diameter. * The table content summarizes the related topics in the 2024 ESC AF guidelines [4].

**Table 3:** General morphology shapes of the left atrial appendage (LAA), radiologic appearances, and correlation with stroke risk [83,86,87].

Morphology	Prevalence	Radiologic Appearance	Correlation with Stroke Risk
Chicken Wing	48%	An obvious bend in the proximal or middle part of the dominant lobe, or folding back of the LAA anatomy on itself at some distance from the perceived LAA ostium.	*Low risk* Narrow and tortuous, thrombus formation is low.
Windsock	19%	There is one dominant lobe of sufficient length as the primary structure. Variations in this LAA type arise with the location and number of secondary or even tertiary lobes arising from the dominant lobe.	*Moderate/high risk* More prone to blood flow stasis and thrombus formation due to the complex and voluminous structure.
Cauliflower	3%	Limited overall length with more complex internal characteristics. Variations in this LAA type have a more irregular shape of the LAA ostium (oval vs. round) and a variable number of lobes, lacking a dominant lobe.	*Very high risk* A complex anatomical structure increases the risk of thrombus.
Cactus	30%	A dominant central lobe with secondary lobes extending from the central lobe in both superior and inferior directions	*Moderate risk* Structural branching creates an intermediate profile for thrombosis.

LAA, left atrial appendage.

## Data Availability

The original contributions presented in this study are included in the article/Supplementary Materials. Further inquiries can be directed to the corresponding author.

## Supplementary Materials

The following supporting information can be downloaded at: https://www.mdpi.com/article/10.3390/jcm15051758/s1, Final Questions for the Panel Meeting.
