# Supplementary material for: Additional Stroke Risk Factors Beyond the CHA2DS2-VA Score in Non-Valvular Atrial Fibrillation: An Interdisciplinary Expert Opinion
Source: J Clin Med. 2026 Feb 26;15(5):1758. doi: 10.3390/jcm15051758 (PMC12985467; doi:10.3390/jcm15051758)
Supplement: Supplementary file 1 [file jcm-15-01758-s001.zip › jcm-4121438-supplementary.pdf]

orThese advisory board questions were developed to explore the clinical relevance and practical implications of additional risk factors, not included in the CHA_2DS_2-VASc score, for thromboembolic event and stroke prevention in patients with nonvalvular atrial fibrillation (NVAF). The questions aim to reflect real-world clinical decision-making, focusing on renal dysfunction, obesity, cardiac structural and functional parameters, biomarker-based risk stratification, and multidisciplinary management approaches in both primary and secondary stroke prophylaxis. The content of these questions served as the framework for the scientific review presented in the main manuscript.

The Role and Importance of Additional Risk Factors Not Included in the CHA_2DS_2-VA Score Scheme in Thromboembolic Event/Stroke Prophylaxis in Patients with Nonvalvular Atrial Fibrillation (NVAF)

In clinical practice, the CHA_2DS_2-VA score scheme is widely used in the risk assessment (stratification) of developing thromboembolic events (stroke) in patients with nonvalvular atrial fibrillation (NVAF).

1. In your daily clinical practice, approximately what percentage of your NVAF patients are low-to-moderate (CHA_2DS_2-VA 0 or 1) and high-risk (CHA_2DS_2-VA $\geq$ 2) patients?

2. What is your decision-making flowchart when deciding on primary stroke prophylaxis, especially in patients in the low-to-moderate risk group?

3. When determining primary prophylaxis for your NVAF patients in the low-to-moderate risk group for stroke, do you include the additional risk factors mentioned in the guidelines in your assessment?

4. In which NVAF patient subgroups should which additional risk factors be assessed and how in preventing thromboembolic events (stroke)?

In our country, renal dysfunction represents a significant comorbidity in patients with NVAF, similar to its prevalence in the general population.

5. What is the distribution of renal dysfunction among NVAF patients in our country according to categories (CKD 1-5)? (CKD 1-5/albuminuria 1-3/GFR level according to NVAF patient age/gender)

6. In daily clinical practice, what should be considered in primary prophylaxis planning, disease management, and patient follow-up in NVAF patients with proteinuria (>150 mg/24 hours or equivalent) and/or eGFR (<45 mL/hour)? (Follow-up criteria, follow-up frequency, additional examinations, what should be considered in the patient's daily life?)

7. In your opinion, what are the shortcomings in the management and follow-up of primary prophylaxis of thromboembolic events/stroke in NVAF patients with renal dysfunction in routine clinical practice in our country?

8. How can the management and follow-up of this patient group be improved? (healthcare levels/physician awareness/regular evaluation of factors affecting treatment success, etc.)

9. Can you describe the NVAF patient profile where joint follow-up by Nephrology and Cardiology disciplines would be beneficial? Can multidisciplinary work be incorporated into daily clinical practice? What can be done?

10. What is your approach in clinical practice for NVAF patients with end-stage renal failure or those undergoing dialysis?

Our country is facing a growing burden of obesity across the life course, from children and adolescents to adults, with approximately one in three individuals affected.

11. What is the obesity paradox in NVAF patients? This paradox What can it lead to in the medium/long term, especially in terms of stroke risk?

12. What can you say about the distribution of the NVAF patient population in our country according to Body Mass Index (BMI)?

13. What are the common problems/clinically neglected points in primary thromboembolic prophylaxis in overweight/obese NVAF patients?

14. What is biomarker-based predictive modeling in NVAF-related stroke risk assessment, and how is it applied? (ABC-AF stroke, NT-proBNP, cardiac troponin) What are the contributions of this modeling to daily clinical practice? What can be done for its routine use in our country?

15. What is the importance of cardiological factors (high NVAF load, NVAF type, NVAF duration) in NVAF follow-up in clinical practice? What do these factors lead to in the medium/long term, especially in primary stroke prophylaxis? How are they managed?

16. In clinical practice, what is the importance of hypertrophic factors in NVAF follow-up? What is the significance of the presence of cardiomyopathy (HCM)? Considering the primary prophylaxis of stroke, particularly, what may HCM lead to in moderate/long-term follow-up? How are patients with HCM+NVAF managed?

17. What is the importance of left ventricular volume or diameter size in disease management in patients with NVAF? What should be considered/how is disease management performed in this patient subgroup in the medium/long term in the primary prophylaxis of stroke?

18. Considering NVAF patients in the context of these risk factors, what is your choice of treatment agent in primary prophylaxis for thromboembolic events/stroke? (DOACs vs. VKA)

In NVAF patients who cannot tolerate long-term oral anticoagulant (OAC) therapy, left atrial appendage (LAA) emptying rate monitoring and closure is recommended as a current treatment option.

19. What is the role of LAA emptying rate monitoring in NVAF management? In which NVAF patient subgroups is LAA closure recommended? (LAAC) is indicated?

20. In NVAF patients, what risk factors and clinical conditions do you consider in secondary thromboembolic event/stroke prophylaxis? What are your follow-up intervals and follow-up criteria in secondary prophylaxis? What are the roles of other medical disciplines in secondary prophylaxis? Why should secondary prophylaxis be applied?
